# Knowledge of HIV status among men aged 20–35 years in Lusaka, Zambia: Findings from a pilot time location sampling survey in the “Yaba Guy Che” (for the guys) study

**DOI:** 10.1111/tmi.14128

**Published:** 2025-05-24

**Authors:** Mwelwa Muleba Phiri, Lucheka M. Sigande, Chisanga Mwansa, Ab Schaap, Sian Floyd, Loyd Kalekanya, Charles Banda, Steve Belemu, Helen Ayles, Musonda Simwinga, Bernadette Hensen

**Affiliations:** ^1^ Zambart Lusaka Zambia; ^2^ Department of Infectious Disease Epidemiology Faculty of Epidemiology and Population Health, London School of Hygiene and Tropical Medicine London UK; ^3^ Department of Clinical Research Faculty of Infectious and Tropical Diseases, London School of Hygiene and Tropical Medicine London UK; ^4^ Department of Public Health Institute of Tropical Medicine Antwerp Belgium

**Keywords:** feasibility, HIV testing, men, time location sampling, Zambia

## Abstract

**Objectives:**

We conducted a pilot time location sampling survey with young men aged 20–35 years in Lusaka, Zambia and aimed to describe knowledge of HIV status and determine factors associated with knowledge of HIV status.

**Methods:**

Hotspots where men congregate were identified in a densely populated community in Lusaka. Hotspots were grouped into five strata (betting shops; car parks/washes; bus stations/taxi ranks; churches; and markets/shopping streets) and day/times when hotspots were frequented by men were listed. Within each stratum, three hotspots were randomly selected. Subsequently, 1 day/time was randomly selected for each hotspot. Men aged 20–35 were approached for participation and data was collected between July and October 2022. We describe participation in the survey, socio‐demographics, and sexual behaviours. Using logistic regression, we explored factors associated with knowledge of HIV status.

**Results:**

339 men were approached, among whom 304 (90%) were eligible and 297 (98%) consenting to participate. Overall, 61% knew their HIV status. Adjusting for recruitment strata, knowledge of HIV status was similar by age (20–24: 56%; 25–29: 68%; and 30–35: 55%; *p* = 0.19). Among men reporting sex in the last month, men reporting no condomless sex were more likely to know their HIV status (78.2%) compared to men reporting one condomless sex partner in the past 1 month (55.5%; age‐adjusted OR = 3.02; 95%CI 1.07, 8.55; *p* = 0.07). Knowledge of HIV status was lower among men who thought their friends were testing every 2–5 years (48%; *n* = 12/29) compared to those assuming that their friends tested more frequently (70.0%; adjOR = 0.28; 95%CI 0.08, 0.98; *p* < 0.001).

**Conclusion:**

The time location sampling survey was acceptable among men, as evidenced by high participation. Overall, 40% of young men did not know their HIV status. A hotspot‐driven approach to delivering HIV testing services may prove effective at reaching men. Furthermore, time location sampling surveys should be explored as a tool to evaluate interventions targeting men.

## BACKGROUND

Globally, HIV incidence has reduced by approximately 28% in Eastern and Southern Africa, since 2010 [1, 2]. Despite this progress, HIV incidence has remained high in many countries in these regions. It is well established that in Eastern and Southern African countries, men are less likely than women to know their HIV status and to have poorer outcomes across the HIV care continuum and prevention cascade. As a result, men have sometimes been described as “hard to reach” with services and labelled as “difficult” to engage [3, 4]. Although men are not a homogenous group, their limited uptake of available HIV services is, in part, attributable to how the services are delivered, being inappropriate, unacceptable or inaccessible to men. Men may also face barriers due to gender norms regarding masculinity, such as feeling that men “should” be considered in control and have knowledge, but to access services, they are expected to show deference or compliance, which are considered “feminine” traits [5, 6, 7]. However, it is becoming increasingly clear that men are willing to test for HIV when services reach them, with community‐based delivery of HIV testing services more likely to reach men than facility‐based services and community‐based spaces reaching high numbers of men [3, 8, 9, 10].

In Zambia, the 2018 Demographic and Health Survey reports that 52% of men tested positive for HIV in the last 12 months compared to 64% of women [11]. The HPTN 071 (PopART) trial, which estimated the impact of universal HIV testing‐and‐treatment on HIV incidence in 21 communities in South Africa and Zambia, found that the intervention had a greater impact on HIV incidence among men than among women, through women's greater engagement with home‐based delivery of universal testing‐and‐treatment services [12]. Recent analysis of phylogenetic data from the HPTN 071 (PopART) trial in Zambia found that men were two times more likely to transmit HIV than women, with approximately a third of transmissions from men aged 25 to 39. In addition, men aged 35–39 years were 5.98 times more likely to transmit HIV than their female peers [13]. This suggests that efforts to prevent transmission, including the use of pre‐exposure prophylaxis, should target this group of men as well as younger men aged less than 25 years old.

Household surveys are generally used to obtain estimates of HIV‐related outcomes among a representative sample of the general population. However, studies have found that men are often not home during these surveys, which are generally conducted during conventional working hours [14, 15]. Consequently, men can be underrepresented in household surveys and/or more resources are required to reach men who are home to reach required sample sizes [14, 15]. Alternative sampling strategies, including respondent driven and time location sampling (TLS) (also known as venue‐day‐time sampling or time–space sampling), are used to conduct surveys with populations that are “hidden” including sex workers and men who have sex with men [16, 17]. Considering men are less likely than women to be found at home during household surveys, these alternative sampling strategies could be effective at reaching men and could be used to estimate health‐related outcomes (including HIV) among mobile men [18].

In this study, we aimed to assess the feasibility of time location sampling to conduct a survey with young men aged 20–35 years residing in a high‐density urban setting in Lusaka, Zambia. We also aimed to describe knowledge of HIV status and determine factors associated with knowledge of HIV status.

## METHODS

### Study location and population

This study was conducted between July and October 2022 in a densely populated, urban community in Lusaka, Zambia with a population of approximately 200,000. The population of interest for the survey was men aged 20–35 years residing in the study community.

### Sampling and sample size

Before the survey, venues (or locations) where men convene in the community were mapped by the study team, who were residents of, or familiar with, the study community. This mapping was restricted to being conducted during specific hours, that is, before 9 pm to ensure the safety of study staff and the vehicle. During focus group discussions, part of a broader formative study, participants were also asked about venues in the community where men convene. The study team systematically documented the name and location of the venues and estimated the number of men frequenting the space as part of the mapping. While bars were cited as venues where men convene, they were excluded from this study due to ethical concerns regarding obtaining informed consent to participate in the study by patrons potentially consuming alcohol and the safety considerations for the study staff.

Once considered completed, venues (or locations) were grouped into two broad categories—named venues and high‐density locations. Named venues were locations such as betting shops, churches, and car washes that could be individually named and visited. High‐density areas were primarily large markets that were to be divided into smaller geographical areas, with each area allocated a number. The list of venues (universe of venues) was subsequently stratified into five groups: churches; car parks, car washes, and garages; taxi ranks, bus stations, and wholesale shops; betting shops; and high‐density areas. This stratification was selected based on the assumption that men visiting the venues within each stratum would be more similar to each other than men visiting venues in other strata. Within each stratum, three venues or numbered areas were randomly selected. To include 16 venues, we randomly selected a stratum and from within this one stratum selected an additional venue.

For all venues, we had information about the day and 4‐h time slots within these days when the venue was busiest, though venues were also visited during off‐peak hours, which allowed us to determine peak and off‐peak hours. For the randomly selected venues, these day/time combinations were listed. For venues with only 1 day/time slot, for example, some churches, this was the selected day/time that the venue was scheduled to be visited by two research assistants (RAs) during the survey. For others, 1 day and 4‐h time slot was randomly selected for each randomly selected venue. These were then scheduled based on the day selected (e.g., Monday scheduled first), considering public holidays. One exception was randomly selected betting shops, with visits scheduled for August to coincide with the football season when venues would be busiest. This was a consideration because the betting was primarily conducted on football matches, in particular, based on English Premier League matches, which would begin in August. Each venue had three data collection days.

Prior to the survey, the RAs would inform the manager, owner, or other responsible individual of the purpose of the study. On the randomly selected day/time slot for each venue, two RAs visited the venue and, for the first 30 min of the 4‐h slot, counted the number of men at the venue who appeared to be in the eligible age range. The RAs approached men considered to be of the appropriate age group and briefly informed them about the study. If men agreed to hear more information and were eligible, they were given detailed information and asked for written informed consent to participate. Consenting men were asked to complete a short questionnaire, which included questions on socio‐demographics, sexual behaviour, knowledge of HIV transmission and prevention, norms regarding HIV testing, history of HIV testing, sexual behaviour, and perceptions regarding the delivery of HIV testing to men.

As a feasibility study, there was no sample size calculation. Rather, the aim was to assess whether men would be willing to participate in a survey outside of household settings, and thus determine whether TLS could be used to evaluate the impact of a future strategy to deliver HIV testing services to young men.

### Outcomes and explanatory variables

The primary outcome of interest was knowledge of HIV status (defined as self‐reporting knowing ones HIV positive status or HIV testing in the past 12 months). Factors explored for their association with the outcome included: educational attainment, employment status, marital status, sexual behaviour (including ever had sex, number of partners in last 12 months, had sex in the last month, and number of (condomless) sex partners in the last month).

### Data analysis

We first described the number of men enumerated at each venue and stratum. Subsequently, we weighted the data as described in the “UCSF Institute for Global Health Science Resource Guide: Time Location Sampling” [19]. Regardless of how many men attended each venue, the number of men recruited was restricted by the 4‐h data collection timeslot. We therefore weighted the data to ensure the men sampled were representative of the men attending each venue. To do this, we first estimated the total number of men observed at each venue by summing the number of men aged 20–35 years observed at each venue during each 4‐h time slot. Next, using the number of men consenting to complete the survey at each venue, we divided the number enumerated by the number consenting to estimate a weight for each venue.

Applying weights, we describe men's socio‐demographics, including their age, highest level of education attained, whether men were currently employed, and how long they resided in the study area. We also describe perceived norms regarding HIV testing, HIV testing behaviours, use of antiretroviral therapy (ART) or pre‐exposure prophylaxis (PrEP), and uptake of voluntary medical male circumcision services. We also describe sexual behaviours as described and present unweighted percentages for comparison. Using logistic regression analysis, and applying survey weights, we explored the factors associated with knowledge of HIV status. Twenty‐seven men reported HIV testing in 2021 but did not know in what month they tested for HIV. In a sensitivity analysis, we included these men as knowing their HIV status (thus assuming they had tested for HIV in the 12 months before the survey).

### Ethics

Written informed consent was obtained from all the participants before the questionnaire was administered. Ethical approval was obtained from The London School of Hygiene and Tropical Medicine (Ref: 26713) and the University of Zambia Biomedical Research Ethics Committee (Ref: 2374‐2021). The study received regulatory approval from the Zambia National Health Research Authority (NHRA).

## RESULTS

**FIGURE 1:**
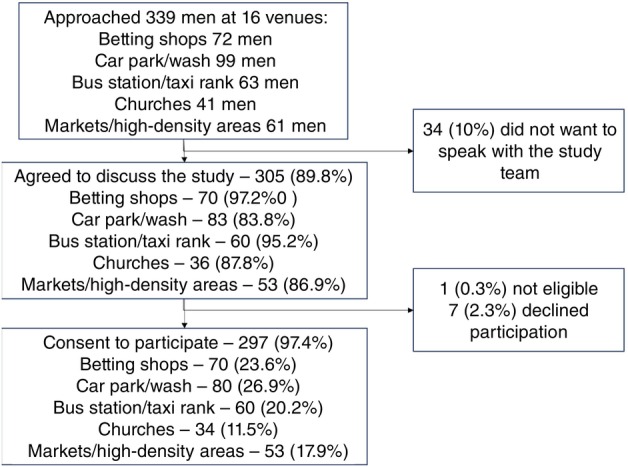
Participation of men aged 20–35 years in the time location sampling survey by venue type (*N* = 297).

Across the 16 venues, 339 men were approached; 90% (*n* = 305) agreed to discuss the study with the RAs. Of these, 304 were eligible and almost all men (297; 97.4%) consented (Figure 1). Slightly more men were recruited from car parks and car washes (26.9%, *n* = 80), betting shops (23.6%, *n* = 70), and bus stations/taxi ranks (20.2%, *n* = 60) than from markets (17.9%, *n* = 53) and churches (11.5%, *n* = 34).

### Sociodemographic characteristics and sexual behaviours

Most of the men were aged 20–29 (*n* = 230; 77.4%), 61% (*n* = 182) reported never being married, and almost all had lived in the study community for >2 years (*n* = 267; 89.9%) (Table 1). The most commonly reported level of education attained was incomplete secondary education (*n* = 112; 37.7%) and two‐thirds of men were currently employed (*n* = 192; 64.2%). Approximately half (*n* = 135; 45.5%) reported living with 3–5 household members and not having a smartphone with access to the internet (*n* = 162; 54.5%).

**TABLE 1:** Sociodemographic characteristics and sexual behaviours of men aged 20–35 recruited through time location sampling, 2022.

Socio‐demographic characteristics	%	Weighted (%)
Age		
20–24	121 (40.7%)	37.4
25–29	109 (36.7%)	39.4
30–35	67 (22.6%)	23.2
Marital status		
Single—never married	182 (61.3%)	59.1
Currently married/living with my spouse	86 (29.0%)	31.2
Currently married but living apart	5 (1.7%)	1.5%
Previously married—divorced or separated	19 (6.4%)	6.6
Previously married—widowed	5 (1.7%)	1.6
Highest level of education		
None/(in)complete primary	60 (20.2%)	20.1
Incomplete secondary	112 (37.7%)	39.7
Complete secondary	104 (35.0%)	34.0
Higher education	21 (7.1%)	6.2
Currently employed		
No	105 (35.4%)	33.3
Yes	192 (64.6%)	66.7
Time lived in community		
Up to 2 years	21 (7.0%)	6.7
2 years	9 (3.0%)	3.2
3+ years	267 (89.9%)	90.1
Whether they own a smartphone		
No	162 (54.5%)	57.5
Yes—have my own	132 (44.4%)	41.8
Yes—shares one with HH member	3 (1.0%)	0.7
Number of household members		
1–2	71 (23.9%)	24.0
3–5	135 (45.5%)	45.0
>5	91 (30.6%)	31.1
Sexual behaviours		
Ever had sex		
No	15 (5.1%)	4.1
Yes	281 (94.9%)	95.9
Number of sex partners in the last month (among men who ever had sex; *N* = 281)		
None—no sex last month	105 (37.4%)	34.5
One	108 (38.4%)	37.8
2–3	48 (17.1%)	20.8
4+	20 (7.1%)	6.8
Number of partners with whom had condomless sex in last month (*N* = 266)^a^		
None or no sex last month	137 (51.5%)	48.6
One	96 (36.1%)	38.2
2–3	33 (12.4%)	11.7
Linkage to HIV prevention services		
Ever taken PrEP (*n* = 291)^b^		
No	279 (95.9%)	95.8
Yes	12 (4.1%)	4.2
Ever circumcised (*N* = 297)		
No	119 (40.1%)	39.9
Yes	178 (59.9%)	60.1

^a^ *n* = 15 did not respond to the question on number of condomless sex partners.

^b^ Restricted to men self‐reporting their HIV negative status or testing HIV positive after PrEP availability in Zambia (2018).

Most men reported ever having had sex (*n* = 281; 94.9%). Among these men, 37.4% (*n* = 105) reported no sex in the past month, 38.4% (*n* = 108) reported 1 partner, and 24.2% (*n* = 68) reported ≥2 partners. Among men reporting any sexual partners in the last month, 54.5% (*n* = 96/176) reported condomless sex with one partner in the last month; 18.8% (*n* = 33/176) reported condomless sex with ≥2 partners.

### Perceptions regarding HIV testing and preferences for HIV testing delivery

**FIGURE 2:**
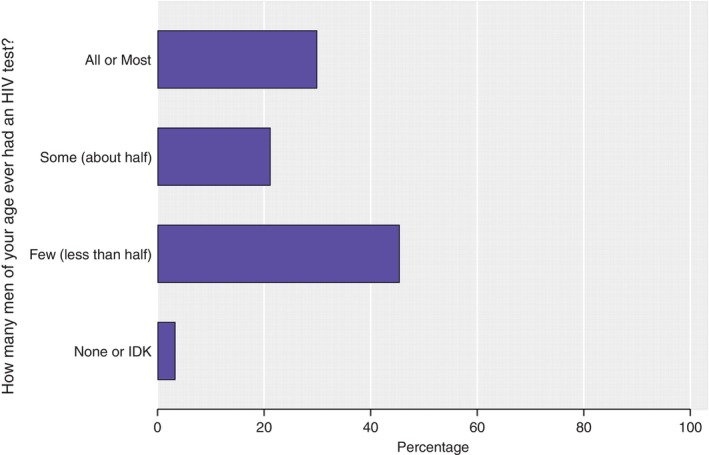
Perceptions regarding HIV testing among male peers by men participating in the survey(*N* = 297).

Although the percentage of men considered it equally important for women and men to test for HIV (99.0%, *n* = 294/297, and 99.0%, *n* = 294/297, respectively), when asked how many of their peers they thought had ever tested for HIV, 45.5% (*n* = 135) thought few (less than half of all men) had ever tested for HIV (Figure 2). Conversely, when asked how frequently they thought their male friends tested for HIV, one‐third thought once a year (35.0%; *n* = 104).

Almost all men agreed that fear of an HIV positive status was why men chose not to test for HIV (*n* = 285; 96.0%), followed by fear of being seen accessing services and therefore being stigmatised/discriminated against (*n* = 252; 84.8%), and not wanting to go to the health facility in general (*n* = 246; 82.8%). The main reason men were thought to test for HIV was to care for their health (*n* = 145; 48.8%), followed by fear of having been exposed to HIV (*n* = 77; 26.0%) and feeling unwell (*n* = 38; 12.18%).

When asked if they would be willing to have an HIV test at the location of recruitment, 95.8% (*n* = 286) of men responded yes. When asked about other locations from which they would be willing to access HIV testing (Table 2), most men were willing to access HIV testing services from a tent (97.3%) or mobile van (93.9%) in their community and at a marketplace (77.1%).

**TABLE 2:** Willingness to test for HIV at listed location among men that participated in the survey (*N* = 297).

Locations	Number (%)	Weighted (%)
Kantemba	126 (42.4%)	42.1
Bar	75 (25.3%)	25.4
Barbershop	121 (40.9%)	38.7
Mobile van	279 (93.9%)	95.5
Tent	289 (97.3%)	98.1
Church	108 (36.5%)	33.3
Betting shops	149 (50.3%)	50.4
Market	229 (77.1%)	76.3
Car wash/garage	137 (46.1%)	45.5

### Levels of and factors associated with knowledge of HIV status

Over half (*n* = 169/292; 60.7%) the men currently knew their HIV status; 6% of these men (6.4%; *n* = 10) self‐reported living with HIV. Almost all men reported ever testing for HIV (*n* = 274; 93.5%).

In our risk factor analysis, we found little evidence of an association between socio‐demographic factors and knowledge of HIV status. Among men reporting sex in the last month, men who did not report any condomless sex were more likely to know their HIV status (78.2%) compared to men reporting one condomless sex partner in the past 1 month (55.5%; adjOR = 3.46 95%CI 1.33, 8.90; *p* = 0.02) (Table 3). There was also evidence of an association with perceived frequency of HIV testing among male friends; knowledge of HIV status was lower among the few men who thought their friends were testing every 2–5 years (48%; *n* = 12/29) compared to those guessing that their friends tested more frequently, specifically once or more than once a year (70%; *n* = 113/168; adjOR = 0.28 95%CI 0.08, 0.98; *p* < 0.001).

**TABLE 3:** Levels of and factors associated with knowledge of HIV status among men aged 20–35 years who participated in the time location sampling survey (*N* = 292).

Socio‐demographic characteristics	Number	Knows HIV status	% (wgt %)	Age‐adjusted OR (95%CI)	Adjusted OR^a^ (95%CI)	*p*‐Value
Age						
20–24	117	61	52.1 (56.1)	1.0	1.0	0.19
25–29	109	70	64.2 (67.9)	1.66 (0.90, 3.04)	1.66 (0.90, 3.04)	
30–35	66	38	57.6 (55.3)	0.97 (0.48, 1.94)	0.97 (0.48, 1.94)	
Marital status						
Single—never married	178	95	53.4 (57.0)	1.0	1.0	0.22
Currently married	90	60	66.7 (69.6)	1.88 (0.87, 4.05)	1.88 (0.87, 4.05)	
Previously married—divorced, separated or widowed	24	14	58.3 (52.3)	1.03 (0.33, 3.25)	1.03 (0.33, 3.25)	
Highest level of education						
None/(in)complete primary	60	30	50.0 (53.7)	1.0	1.0	0.45
Incomplete secondary	109	62	56.9 (58.7)	1.19 (0.57, 2.49)	1.19 (0.57, 2.49)	
Complete secondary	102	65	63.7 (67.3)	1.77 (0. 84, 3.76)	1.77 (0. 84, 3.76)	
Higher education	21	12	57.1 (59.8)	1.28 (0.43, 3.84)	1.28 (0.43, 3.84)	
Currently employed						
No	105	55	53.9 (55.5)	1.0	1.0	0.35
Yes	190	114	60.0 (63.3)	1.31 (0.75, 2.28)	1.31 (0.75, 2.28)	
Number of household members						
1–2	71	40	56.3 (57.7)	1.0	1.0	0.83
3–5	133	80	60.2 (62.8)	1.23 (0.63, 2.41)	1.23 (0.63, 2.41)	
>5	88	49	55.7 (60.0)	1.18 (0.56, 2.49)	1.18 (0.56, 2.49)	
Sexual behaviours						
Had sex in the last 12 months						
No—or never had sex	118	61	51.7 (52.7)	1.0	1.0	0.15
Yes	173	107	61.9 (64.7)	1.56 (0.85, 2.86)	1.56 (0.85, 2.86)	
Number of sex partners in the last month (among men who ever had sex; *N* = 277)						
None—no sex last month	104	53	51.0 (52.7)	1.0	1.0	0.21
One	105	62	59.1 (62.0)	1.39 (0.71, 2.71)	1.39 (0.71, 2.71)	
2–3	48	30	62.5 (64.2)	1.51 (0.66, 3.46)	1.51 (0.66, 3.46)	
4+	20	15	75.0 (80.8)	3.51 (1.08, 11.5)	3.51 (1.08, 11.5)	
Number of condomless partners in the last month (*N* = 158)^b^						
None	31	23	74.2 (78.2)	3.02 (1.07, 8.55)	3.02 (1.07, 8.55)	0.07
One	94	49	52.1 (55.5)	1.0	1.0	
2+	33	23	69.7 (73.6)	2.12 (0.75, 5.95)	2.12 (0.75, 5.95)	
Perceptions related to HIV testing behaviours^c^						
People in my community think it is important for men to test for HIV						
Agree	57	31	54.4 (56.8)	0.79 (0.40, 1.56)	0.80 (0.40, 1.61)	0.12
Disagree	225	133	59.1 (62.5)	1.0	1.0	
I don't know	10	5	50.0 (40.4)	0.39 (0.10, 1.59)	0.22 (0.05, 0.97)	
Male peers in my community think it is important to test for HIV						
No	48	25	52.1 (53.0)	1.0	1.0	0.50
Yes	244	144	59.0 (62.3)	1.39 (0.69, 2.79)	1.28 (0.62, 2.65)	
How many men of your age do you think have ever tested for HIV						
All or most (at least more than half)	88	54	61.4 (65.9)	1.0	1.0	0.22
Some (about half)	61	39	63.9 (68.3)	1.19 (0.56, 2.54)	1.47 (0.66, 3.30)	
Few (less than half)	133	71	53.4 (55.1)	0.66 (0.35, 1.25)	0.75 (0.38, 1.47)	
I don't know/none	10	5	50.0 (38.9)	0.34 (0.07, 1.61)	0.43 (0.10, 1.87)	
How often do you think your male friends test for HIV?*						
Once a year or more than once a year	168	113	67.3 (70.0)	1.0	1.0	0.01
Once every 2–5 years	29	12	41.4 (48.0)	0.39 (0.15, 1.01)	0.51 (0.18, 1.39)	
Only once a lifetime	16	8	50.0 (40.9)	0.29 (0.09, 0.89)	0.23 (0.06, 0.74)	
I don't know/never	79	39	45.6 (50.0)	0.42 (0.23, 0.79)	0.39 (0.20, 0.78)	

^a^ Further adjusted compared to age‐ and strata adjustment only where reported.

^b^ 15 men did not respond to the condomless sex question.

^c^ Adjusted for number of condomless sex partners in last month (with men reporting no sex in the last month coded as none (0)).

In our sensitivity analysis, 66.7% (*n* = 196) of men knew their HIV status. Results of the risk factor analysis were similar; however, there was less evidence for an association between knowledge of HIV status and perceived frequency of friends' HIV testing (*p* = 0.09).

### Coverage of HIV prevention and treatment services

Forty‐one percent of men (40.7%; *n* = 121) had heard of PrEP, among whom 10.0% (*n* = 12) reported ever taking PrEP. Sixty percent (*n* = 178; 60.0%) of men reported being circumcised, among whom 52.5% reported being circumcised to protect themselves from HIV; 20.4% reported hygiene‐related reasons, and 16.3% reported that they were circumcised for traditional reasons.

## DISCUSSION

The conduct of a TLS survey to reach young men in a densely populated community in Lusaka was feasible, with 90% of men approached consenting to participate in the survey and almost 300 men completing the survey. Among the age group of interest, a higher percentage of men aged under 30 years were reached; almost all men reported ever testing for HIV and almost two‐thirds currently knew their HIV status. Among this relatively narrow age group, we found few socio‐demographic and behavioural factors associated with knowledge of HIV status. The few men who thought their friends were testing infrequently were also less likely to know their HIV status, suggestive of an influence of perceived HIV testing norms on HIV testing behaviour.

Our study team comprised only two RAs and a community engagement officer; nonetheless, it was feasible to conduct a TLS survey. Although we cannot estimate response to the survey among all men who were present at the venue, as not all eligible men were approached, we found high participation among men approached. In our survey, a higher percentage of men reached were aged 20–24 and 25–29, with 77% of all men falling into these two age groups. In the Zambian 2018 Demographic and Health Survey (DHS), the age distribution of men reached in Lusaka was similar across the three age groups (20–24, 25–29, and 30–35) [11]. In the HPTN 071 (PopART) trial, being absent during household visits increased with age among men aged 20–35 [20]. Taken together, these estimates suggest we reached a lower percentage of the underlying population of men aged 30–35 years than men aged 20–29 years. Compared to the 2018 DHS, a lower percentage of men in our survey reported any employment (84% vs. 65%, respectively) and higher educational attainment (16.7% vs. 7.4%, respectively) [11]. Although challenging to compare as there is no data on socio‐demographics of men who did not participate in the DHS (although response rates were high) and the surveys were conducted at different timepoints and representative at national level, these findings suggest that the men in our sample differ from those reached through household surveys. This is similar to data from community hubs (with HTS) that were implemented after the door‐to‐door HTS provision in the HPTN 071 study in Zambia, which found that the hubs could reach a different sub‐group of men who were not found at home during the household HTS and were younger and likely in informal employment [3]. Despite these findings, additional research with larger study teams and a larger sample of men is required to better understand who is reached through TLS surveys. Nonetheless, the strategy is appropriate to evaluate the impact of community‐based interventions to reach men in community settings; TLS surveys have previously been used in Zimbabwe to estimate the impact of a peer‐network intervention on HIV risk among men attending beer halls [19], and have also been used successfully in the United States in low income communities to explore multi‐level barriers to HIV testing among heterosexual African‐American/Hispanic men [18].

Barriers to HIV testing reported in this study included fear of HIV testing, fear of stigma and discrimination of being seen at the health facility, and not wanting to go to the health facility; this is consistent with numerous studies conducted in Sub‐Saharan countries as reported in a systematic review on barriers and enablers to HIV testing [21]. This is also consistent with findings from the larger formative study in this community that was conducted before the TLS survey [7]. Facilitators to HIV testing reported in this survey were again similar across the systematic review and the formative work and included feeling unwell, fear of HIV exposure, and caring for their health.

Approximately two‐thirds of men in our survey knew their HIV status. In the 2018 Zambian DHS, approximately 64% of men aged 20–35 living in Lusaka tested for HIV in the last 12 months [11]. In a recent cluster randomised trial (CRT) of community‐based, peer‐led SRH services, 51% of men aged 20–24 in 2019 who resided in the control arm and participated in a survey reported knowledge of their HIV status [22]. Results were similar in an analysis of the 2016 South African DHS [23]. Although ever testing was almost universal, the evidence that men aged 25–40 years account for around 40% of male–female HIV transmissions and that this age group reports having multiple partners and persistent condomless sex means it remains critical to support annual HIV testing for men in high HIV burden settings [11, 13]. Community‐based delivery of HIV testing services has demonstrated success in reaching men in high HIV burden settings [3, 8, 22, 24], and such delivery should be considered to reach this population of men in a bid to meet 2030 targets of ending AIDS [25].

In our risk factor analysis, perceived frequency of HIV testing among friends was associated with knowledge of HIV status. This finding is similar to a study conducted in Tanzania, which found that men's perception of HIV testing was clustered within social networks [26]. Similarly, a survey conducted in Uganda found that 51% of men did not consider testing for HIV to be normative despite evidence that over half of all individuals participating in the study had ever tested for HIV [27]. In addition to norms related to HIV testing behaviour, studies have shown that masculine norms affect men's HIV testing behaviour [28]. Behaviours were influenced by masculine norms such as strength, independence, and gendered communication, such as where discussions with women about sex were found to be barriers to uptake of testing, but speaking about sex with other men was deemed acceptable, and this can be leveraged to encourage HIV testing if it is among men [28]. With community‐based HIV testing services, reaching men will require delivery of normative information as a strategy to provide men with accurate information on HIV testing among men in their communities.

Our study has strengths and limitations. Study procedures were rigorous and included a triangulated thorough identification of venues by men living in the community through focus group discussions and by mapping of the community by RAs who themselves lived and previously worked in the community. In addition, we were not denied access to venues randomly selected, which can be a limitation with TLS surveys. However, the men sampled may not be representative of all men in the community, thus introducing a sampling bias, as not all men attend the venues, and some men may work outside of the community (particularly men in formal employment) and therefore be more likely to be missed by a TLS survey. Our estimate of the number of men aged 20–35 at the venues and potentially eligible for the study (that is, our denominator) may be inaccurate as it was challenging to determine the age of men solely through observation. To address this concern, we had two people counting men; their estimates were generally consistent. In addition, the study may have overrepresented young men compared to the population of young men absent from home in the HPTN 071 study, and there may have been social desirability in the reporting of willingness to HIV testing, especially as some participants reported assuming an HIV test would be offered as part of the study. Regardless, this approach is considered appropriate if one wants to reach men, but does require extensive mapping (which may require substantial resources compared to respondent driven sampling for example) and further research needs to be conducted to explicitly compare those absent during HH visits with those participating in TLS.

Overall, 40% of men did not know their HIV status, and only 40% of men had heard of PrEP, highlighting current gaps in HIV testing and prevention information not reaching men aged 20–35 who are key to achieving control of the HIV epidemic and ending AIDs among this population. These results point towards the need for a hotspot‐driven approach to delivering HIV testing services that may prove effective at reaching this group of men. Lastly, TLS surveys should be explored as a less expensive tool compared to large household‐based surveys and so requiring fewer resources, to evaluate interventions targeting men in this hotspot‐driven approach with a larger sample size to allow for a more in‐depth understanding of men reached through this method. However, further research to determine the costs of conducting a TLS for evaluation in this population would be recommended to inform consideration of its use on a larger scale.

## CONSENT FOR PUBLICATION

All individuals provided consent to publish anonymised information.

## AUTHOR CONTRIBUTIONS

MMP and BH wrote the original draft of the manuscript. BH led the formal analysis with input on interpretation from MMP and SF. AJS and LMS were responsible for the software and data curation. MMP and SB led the project administration, supervision, and investigation. CM, MS, CB, and LK were involved in the investigation. BH, MMP, and HA were involved in the conceptualisation, methodology, funding acquisition, project administration, and supervision. All authors were involved in the design of the study, contributed to the writing, reviewing, editing of the paper, and read and approved the final version.

## FUNDING INFORMATION

This research was jointly funded by the UK Medical Research Council (MRC) and the Foreign Commonwealth and Development Office (FCDO) under the MRC/FCDO Concordat agreement, together with the Department of Health and Social Care (DHSC) (Grant number MR/V031171/1). The funders had no role in study design, data collection and analysis, decision to publish, or preparation of the manuscript.

## CONFLICT OF INTEREST STATEMENT

The authors declare that they have no competing interests.

## Data Availability

The datasets analysed for the current analysis are available from the corresponding author on reasonable request.
